# Development of cereal and legume based food products for the elderly

**DOI:** 10.1186/2193-1801-3-451

**Published:** 2014-08-20

**Authors:** Pruet Satusap, Visith Chavasit, Wantanee Kriengsinyos, Kunchit Judprasong

**Affiliations:** Institute of Nutrition, Mahidol University at Salaya, Nakhonpathom, 73170 Thailand

**Keywords:** Elderly, Cereal, Legume, Energy distribution, Starch *in vitro* digestion

## Abstract

Diets for elderly must contain nutritious foods, fit their physiological limitations, and match with their food culture. Cereals and legumes are suggested food choices regardless of their cultures and beliefs. Ready-to-eat products containing suitable macronutrient patterns from cereals and legumes were developed. Energy distributions from carbohydrate (60 kcal/100 kcal), protein (15 kcal/100 kcal), and fat (25 kcal/100 kcal), protein quality, and percent energy from saturated fatty acid and free sugar were criteria for the formulation. Carbohydrate sources were rice flour, brown rice flour, mung bean starch, which carbohydrate in rice flour was the most digestible on *in vitro* test. Protein and fat sources were soybean flour, black sesame seed, and rice bran oil. Three products, i.e., flake snack, instant beverage, and instant soup were produced by drying basic ingredients as flakes on a double-roller drum dryer and directly used or dry-mixed with other ingredients. The products (Aw <0.3) had balanced energy distribution, good quality protein, and energy from saturated fat < 8 kcal/100 kcal and free sugar < 10 kcal/100 kcal. Results from sensory central location test in 219 elderly subjects indicated that the flake snacks from both carbohydrate sources were significantly more acceptable than the other two products.

## Introduction

Elderly are becoming a large proportion of the world’s population in developed and developing countries, increasing from 0.6 billion in 2000 to 2 billion by 2050 (World Health Organization 2002a). The health care costs for this growing age group will likewise increase, especially for curative care, even though prevention is recognized as more economical and sustainable (Chernoff 2006). In this context, nutrition is one of the key elements of success in a health promotion strategy (Morley and Thomas 2007).

Available food products in the market may not be suitable for the elderly in terms of nutrition, physical characteristics, and cost. To prevent elderly persons from becoming under-nourished and reduce the risk of non-communicable diseases, their meals should have a balanced energy distribution in terms of macronutrients as well as other factors, i.e., carbohydrates of low glycemic index, minimal added sugar, adequate quality protein, healthy fatty acid ratio, and adequate potentiallydeficient micronutrients (World Health Organization 2002b). Moreover, the physical characteristics of the food should be suitable for elderly persons’ physiological limitations, which usually center on the senses, mastication, and digestion (Chernoff 2006). In addition, food for elderly persons should be affordable and, as such, cereal and legumes are more appropriate choices. Cereals as the main staple foods are widely grown and known as good sources of nutrients. Moreover, the guidelines for healthy living of many countries suggest increased consumption of pulses and nuts (World Health Organization 2014). By combining these plant foods in the right proportions, nutritious foods for the elderly that are acceptable and affordable could be developed for promoting the health and wellness of this growing population in many developing countries (World Health Organization 2002a). In addition, the developed plant-based diets should be easily accepted by elderly persons living in a variety of cultures and holding varying beliefs. Consequently, this study aimed to develop ready-to-eat cereal and legume-based products for the elderly that provided suitable nutrients from cereal and legumes.

## Materials and methods

### Ingredients

Rice flour (Erawan™ brand, Cho Heng Vermicelli Factory Ltd., NakhonPathom, Thailand), brownrice flour (Luk Tao™ brand, Chiensiri Nutritious Food Ltd., Bangkok, Thailand), mung bean starch (Pinebrand, Sitthinan Ltd., Bangkok, Thailand), soybean flour (DoiKhambrand, Doi Kham Food Products Ltd., Bangkok, Thailand), roasted-ground black sesame (Xongdur™ brand, Xongdur Thai Organic Food Ltd., Nonthaburi, Thailand), rice bran oil (King™ brand, Thai Edible Oil Ltd., Samutprakan), Thailand), shitake-flavored soup powder (FaThaiTM brand, F-Plus Ltd., NakhonnPathom, Thailand), refined sugar granules (MitrPhol™ brand, MitrPhol Sugar Corp., Bangkok, Thailand) and acesulfame K (Xining Pharmaceutical, Xining, China) were obtained from local department stores or supplier in Bangkok, Thailand. Raw materials from different retail packages were pooled and homogenously mixed and repacked under vacuum in nylon-lined plastic bags for powders and in polyethylene terephthalate (PET) bottles for oil. The repacked and mixed materials were stored in cold room at 8°C until use.

### Formulation

The formulation aimed for products that had an energy distribution of carbohydrate, protein, and fat at 60, 15 and 25 kcal/100 kcal, respectively. Their protein qualities should be comparable to requirements set in the latest WHO technical report (World Health Organization 2007). Energies from saturated fatty acid and free sugar were less than 8% and 10%, respectively (World Health Organization 2002b). The carbohydrate sources should be low on the glycemic index. The product prototypes included: (i) flake snack, (ii) instant beverage, and (iii) instant soup. All ingredients, except for rice bran oil, sugar, and acesulfame K, were analyzed for protein, fat, carbohydratecontents and amino acid profile. As carbohydrate sources, rice flour, brown rice flour, and mung bean starch were cooked on electric double-sided flat metal pans in order to simulate cooking with a drum dryer, and then used in the *in-vitro* carbohydrate digestibility test. To combine data from the analyses and nutrient data base (Puwasatien et al. 1999), the formulation was performed by using the mass-balance equation.

### Production

Dried ingredients in the right proportions for each product were mixed with water at the solid content of approximately 40 g/100 g. The slurry was then applied onto a pilot-scale double-roller drum dryer (B.W.S Trading Limited Partnership, Bangkok), 30 cm diameter, 45 cm length, a hard chrome material on the roller surface, with 0.1 mm roller gap, 1 rpm roller speed and 6–9 kg/cm^2^ steam pressure.

The drum-dried sheets of different formulas were broken into approximately 1 cm size and used directly for the flake snack, or dry-mixing with other ingredients i.e. soybean flour, sugar, and acesulfame K for the beverage, and shitake-flavored soup powder for the soup. The products were packed in heat-sealed polypropylene bags until use. The finished products were analyzed for physical, chemical, and sensory qualities, as well as calculated for added sugar.

### Quality analyses

#### *In vitro*carbohydrate digestibility test

This analysis was adapted from the rapid method of monitoring the released glucose during *in vitro* digestion using a hand-held glucometer (Sopade and Gidley 2009). Sample was digested with porcine α-amylase (Sigma A-3176) and then pepsin (Sigma P-6887) before neutralized with 0.2 mol/L NaOH. At pH 6.0, the digestion was continued with pancreatin (Sigma P-1750) and amyloglucosidase (Sigma A-7420). During the digestion period, 6 μl of the digesta was taken at 12 points in time for glucose analysis by using a disposable testing strip attached with an AccuChek® Performa glucometer (Roche Diagnostics (Thailand) Ltd., Bangkok, Thailand). Results read on the test-kit (as mg glucose per dl digesta) were calculated as digested starch and plotted against the sampling time, which later was calculated for area under curve, AUC (Sopade and Gidley 2009).

#### Physical quality

- Water activity
  Water activity was determined on a water activity meter (Novasina MS1 AW, Novasina AG, 8853, 118 Lachen, Switzerland) at 25 ± 1°C.

- Bulk density
  Five gram of flakes was placed in a 50 ml graduated cylinder, gently shaken, and then the volume was read. The bulk density was calculated asfive/volume and recorded as g per ml (Arpanantikul 1999).

- Water holding capacity
  0.50 g of flakes was added into 5 ml of water in a 10 ml graduated cylinder, left till stabilized, and recorded the volume as% volume increase from 5 ml (Elkhalifa et al. 2005).

#### Chemical quality

- Proximate analysis
  Protein was analyzed using the Kjeldahl method (AOAC 2005; 990.20) and content was calculated using the following conversion factors, i.e., 5.71 for soybean, 5.3 for mung bean, 5.95 for rice and brown rice flours, and 6.25 for black sesame seed and shitake-flavored soup powder. Total fat content was determined after acid hydrolysis and solvent extraction using a Soxtec™ apparatus (AOAC 2005; 920.06 C). Ash content was analyzed by heating a sample in a muffle at 550°C (AOAC 2005; 930.30). Moisture content was determined by drying a sample in a hot-air oven at 105°C (AOAC 2005; 990.19). By subtracting the contents of mentioned nutrients from 100, carbohydrate content (including dietary fiber) was determined. The analyses were performed in duplicates based on the procedures of the Association of Official Chemists (AOAC 2006). The energy was calculated by using multiplication factors: 4 for carbohydrate and protein, and 9 for total fat.

- Fatty acid profile
  The fatty profile was analyzed by using gas chromatography after the sample had been acid hydrolyzed and extracted with chloroform (AOAC 2005; 963.22 and 969.33).

- Amino acid profile
  Amino acid profile was analyzed by using liquid chromatography with the precolumnphenylisothiocyanatederivatization method (Hagen et al. 1989).

#### Sensory quality

The finished products were tested for consumer acceptability by using the central location technique (Stone et al. 2012) among 219 male and female elderly volunteers aged 60 years old or above who were obtaining services from the sub-district health promoting hospitals, senior citizen club, and elderly foster home of Phutthamonthon district and nearby, Nakhonpathom province, Thailand. The study was designed as a balance incomplete block, which fixed 3 samples/subject from a total of 6 samples (3 types of product with 2 carbohydrate sources). Each sample was coded with a 3-digit random number and was served to each subject in monadic sequence. Ten grams of the flake snack was served in a capped transparent plastic cup. Ten grams of instant beverage was dissolved in 80 ml of hot water and thenserved in a disposable paper cup. For the instant soup, 5.5 g was mixed with 75 ml of hot water and then served in a disposable bowl with a spoon. A five-point smiley scale was used wherein 1 = dislike very much, 3 = neither like nor dislike, 5 = like very much (Stone et al. 2012). Due to eye sight and literacy problems, each subject was face-to-face interviewed on his/her opinion concerning the products.

### Statistical analysis

The sensory acceptability data was analyzed by using the Statistical SPSS™ software packagefor Windows version 13.0 (SPSS Inc., Illinois, USA). The significant difference at *p* = 0.05 was determined using one-way analysis of variance (ANOVA) and Duncan’s multiple comparison (Stone et al. 2012).

## Results and discussion

### Ingredients

**Figure 1:**
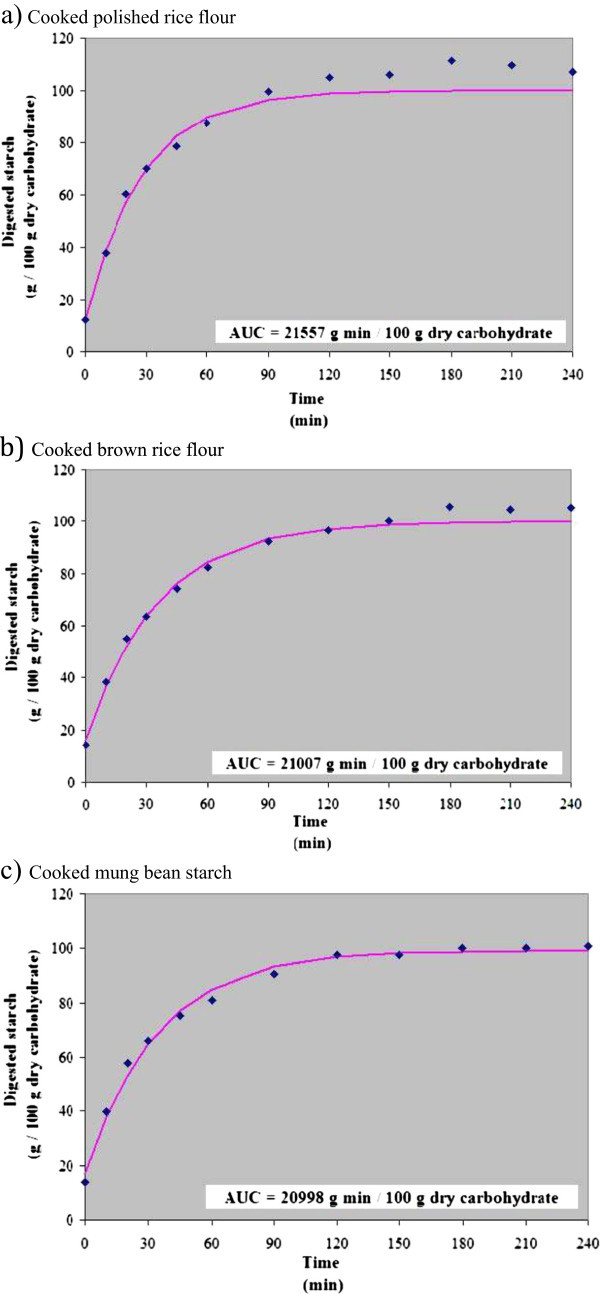
**Digestogram plots and areas under curve (AUC) of the cooked carbohydrate sources: a) Cooked polished rice flour; b) Cooked brown rice flour; c) Cooked mung bean starch (**⧫**: experimental-digested starch;** ^**__**^ **: predicted-digested starch).**

Different plant-based sources of macronutrients in powdered form (i.e., starch and flour) available in the market were chosen, which allowed industries to conveniently access raw materials for producing snacks and instant products. Plant-based materials also provide non-nutrient substances that have physiological functions in the human body (Puwasatien et al. 1999). In addition, most plant-based diets can be consumed by and are affordable to elderly persons living in a variety of cultures and holding various beliefs. Table 1 shows the nutritive values of the plant-based ingredients that were selected for formulation in this study. Rice and brown rice, as sources of carbohydrates, can be different in glycemic index (GI) (Food and Agriculture Organization 1997). For many elderly persons, diabetes mellitus type 2 could be a health problem requiring low GI diets (World Health Organization 2003). Our *in vitro* test on carbohydrate digestibility showed that the brown rice flour had a slower digestibility rate than the polished rice flour (Figure 1). Consequently, brown rice flour should have a better potential for lowering GI. Mung bean starch showed the same trend as the brown rice flour in the *in vitro* study (Figure 1). In fact, mung bean starch has been reported for its good GI in human studies (Juliano et al. 1989, Tangsermwong 2005). Hence, mung bean starch was also a good carbohydrate choice for elderly persons. Compared to other legumes, the soybean was a more interesting source of protein due to its high protein content, lower cost, and wide availability. Soybeans are also a source of fat, especially polyunsaturated fatty acid (Hammond et al. 2003). Black sesame seed was selected as a source of methionine, which was found lacking in the soybeans and cereals (Table 1). Black sesame seed also contains polyunsaturated fat. Rice bran oil was an additional source of fat as well as monounsaturated fatty acid (MUFA), which was found to be too low in the two mentioned fat sources (Puwasatien et al. 1999).

**Table 1:** **Nutritive values of all ingredients and contents of indispensable amino acids of main ingredients used in the formulation**

Ingredient	Composition (g/100 g wet basis)									Energy (kcal)	
	Carbohydrate			Protein			Fat				
Mung bean starch	89.10			0.30			0.00			357.80	
Rice flour	81.70			7.00			0.60			359.70	
Brown rice flour	81.20			8.50			2.60			382.20	
Soybean flour	34.00			35.10			22.40			477.80	
Ground black sesame seeds	17.30			20.50			53.90			635.70	
Soup seasoning	50.94			1.91			3.17			239.87	
**Ingredient**	**Indispensable amino acid (mg/g protein)**										
	**His**	**Ileu**	**Leu**	**Lys**	**Met**	**Cys**	**Met + Cys**	**Phe + Tyr**	**Thr**	**Try**	**Val**
Rice flour	26	31	76	36	14	42	55	100	33	28	46
Brown rice flour	24	28	70	33	12	38	50	85	31	25	42
Soybean flour	29	43	78	64	11	26	37	88	42	18	44
Ground black sesame seed	25	37	66	25	27	29	56	78	36	18	40
Requirement^1^	15	30	59	45	16	6	22	38	23	6	39

^1^From WHO/FAO/UNU, 2007.

### Formulation

Drum-drying is an appropriate process for producing food for the elderly, since it can result in a ready-to-eat and shelf-stable product with suitable physical characteristics (Vijaya et al. 2007). In addition, most drum dried products are produced using plant-based materials, which also is in line with the recommendation to increase consumption of pulses and nuts (World Health Organization 2014). Compared to other preservation techniques, drum drying is not complicated and is not costly. Available drum-dried products in the market include instant cereal beverages. Other traditional crispy cereal sheets and flakes are also available, which are produced by cooking and drying on heated metal plates (similar in concept to drum drying).

This study centered on three product types, namely, a flake snack, an instant beverage, and an instant soup. From assessing the ingredient lists on labels of the commercial flake snacks and instant cereal beverages, it was found that none of them had an appropriate nutrition profile (unreported data). Consequently, the formulation in this study emphasized a healthy nutrient profile as recommended by WHO (World Health Organization 2002b, 2003, 2014).

Table 2 shows the percentages of different ingredients that were used in the different formulas. There were two carbohydrate sources used for all three products, i.e., (i) mung bean starch, and (ii) a mixture of polished and brown rice flours. Mung bean starch by itself showed good AUC output in the *in vitro* test (Figure 1), as well as an ability to form a smooth sheet that was easily removed after cooking on a heated plate (primary trial: unreported). This characteristic highlighted the technical feasibility of using this flour as the main ingredient for a drum-dried product. Brown rice flour had a similar AUC output as the mung bean starch. However, it failed to form an acceptable sheet on the heated plate during sample preparation, unlike polished rice flour. Consequently, both rice flours were mixed in order to improve the AUC output of the polished rice flour and to increase feasibility in drum drying production of the brown rice flour. The mixture of rice flours also contributed a certain amount of protein to the formula, while mung bean starch could not. The main sources of protein for all formulas were soybean flour and ground black sesame seed, which was added to fortify methionine. Both protein sources also contributed a significant amount of fat. However, when the level was still inadequate, rice bran oil was used as an additional source. Sugar was added when necessary to improve sensory quality, though itsuse was limited by using the intense sweetener acesulfame K. Certain ingredients—i.e., soybean flour and sugar in the instant cereal beverage, and the shitake-flavored soup powder in instant cereal soup—were not initially added into the ingredient slurries for drum-drying, since these ingredients were traditionally supposed to be dissolved into the liquid phases of the products. Therefore, they had to be dry-mixed into the drum-dried flakes later on.

**Table 2:** **Percentages of ingredients used in the formulation and serving size of each product**

Product formula ^1^	Snack		Drink		Soup	
	MS	RS	MD	RD	MSO	RSO
Mung bean starch	51.55	-	45.37	-	41.1	-
Rice flour	-	31.16	-	26.67	-	24.79
Brown rice flour	-	31.16	-	26.67	-	24.79
Soybean flour	38.32	25.67	39.96^2^	28.54^2^	32.06	22.31
Ground black sesame seed	4.56	4.58	4.01	4.07	4.11	4.13
Acesulfame-K	0.1	0.1	0.1	0.1	-	-
Rice bran oil	0.91	2.75	1.05	2.44	1.23	2.48
Sugar	4.56	4.58	9.51^2^	9.51^2^	-	-
Soup seasoning	-	-	-	-	21.5^2^	21.5^2^
Serving size^3^	30 g		20 g (160 ml)		22 g (300 ml)	

^1^Main carbohydrate sources i.e. M: mung bean starch, R: polished rice + brown rice flours;

Kinds of product i.e. S: snack flake, D: instant drink, SO: instant soup.

^2^Added during dry-mixing step.

^3^Value in the bracket is the serving size after the product was recombined with water.

### Product qualities

#### Physical properties

Table 3 shows that the mixture could be drum-dried into flakes with different physical characteristics. All flakes had water activity of much lower than 0.6, which could very well inhibit microbial growth (Lenovich 1992). The flakes that used mung bean starch (M) as the carbohydrate source were more bulky (less weight per volume) than the flakes that used rice flours (R). The differences in the nature of carbohydrates as well as other natural nutrients in both carbohydrate sources (M and R) were factors that caused differences in bulkiness and water holding capacity. The formula with mung bean starch also had a higher water holding capacity capacity of about 2-3% as compared with the ones with rich flours (Table 3). The higher water-holding capacity can result in better swallowing for elderly. The powdery characteristic of these products is suitable for elderly who has chewing problem, while the small particle size also increases in surface area that allowed the food to be more expose to enzymes and enhance digestibility.

**Table 3:** **Physical properties** ^**1**^ **of the drum-dried flakes to be used as snack flake (MS, RS), and dry-mixed with other ingredients (before mixing) to produce instant drink (MD, RD) and soup (MSO, RSO)**

Formula ^2^	Aw	Average bulk density (g/ml)	Water holding capacity (% volume increase)
MS	0.287	0.154	16
RS	0.258	0.247	13
MD	0.263	0.126	16
RD	0.267	0.185	14
MSO	0.274	0.175	16
RSO	0.343	0.223	14

^1^Average from 3 samples.

^2^Main carbohydrate sources i.e. M: mung bean starch, R: polished rice + brown rice flours,

Kind of product i.e. S: snack flake, D: instant drink, SO: instant soup.

#### Nutritive values

As the flakes became products, their macronutrient contents and energy distribution were close to the designed values (Table 4). As shown in Table 2, part of the carbohydrate was from the added sugar (sucrose) in all products, however, the energy contributed was only 4.3-8.9 kcal/100 kcal, which was still less than 10 kcal/100 kcal (World Health Organization 2003).

**Table 4:** **Nutritive values (g/100 g), energy distribution (kcal/100 kcal), indispensable amino acid profiles and fatty acid profiles of the finished products**

Formula ^1^	Nutrient content (Energy distribution)										
	Carbohydrate ^2^			Added sugar ^3^			Protein ^4^			Fat	
MS	64.1			4.6			16.1			12.8	
	(58.8)			(4.3)			(14.7)			(26.4)	
RS	65.7			4.6			16.2			12.2	
	(60.2)			(4.3)			(14.8)			(25.0)	
MD	64.0			9.5			15.6			11.9	
	(60.2)			(8.9)			(14.7)			(25.1)	
RD	66.0			9.5			16.1			11.6	
	(61.1)			(8.9)			(14.2)			(24.0)	
MSO	57.4			4.7			14.2			10.9	
	(59.7)			(4.9)			(14.8)			(25.5)	
RSO	58.0			4.7			14.4			11.1	
	(59.5)			(4.8)			(14.8)			(25.7)	
**Formula** ^**1**^	**Indispensable amino acid (mg/g protein)**										
	**His**	**Ileu**	**Leu**	**Lys**	**Met**	**Cys**	**Met + Cys**	**Phe + Tyr**	**Thr**	**Try**	**Val**
MS	38	39	75	59	17	20	37	86	44	21	40
RS	34	35	75	50	15	20	35	89	43	18	40
MD	26	29	73	56	14	17	31	74	38	11	31
RD	26	26	69	46	16	18	34	72	34	12	30
MSO	26	27	70	53	14	17	31	71	36	15	29
RSO	25	27	70	47	13	18	31	71	35	12	31
**Requirement** ^**5**^	**15**	**30**	**59**	**45**	**16**	**6**	**22**	**38**	**23**	**6**	**39**
**Formula** ^**1**^	**Fatty acid (g/100 g)**										
	**Myristic (C14:0)**	**Palmitic (C16:0)**	**Stearic (C18:0)**	**Palmitoleic (C16:1)**	**Oleic (C18:1)**	**Linoleic (C18:2, n-6)**		**γ-Linolenic (C18:3, n-6)**	**S:M:P** ^**6**^		
MS	0.04	1.27	0.50	0.02	2.89	5.11		0.63	1.0:1.6:3.2		
RS	0.06	1.38	0.41	0.00	2.97	4.17		0.42	1.0:1.6:2.5		
MD	0.05	1.10	0.42	0.00	2.45	4.42		0.56	1.0:1.6:3.2		
RD	0.05	1.32	0.40	0.00	2.78	4.21		0.44	1.0:1.6:2.7		
MSO	0.05	1.18	0.42	0.00	2.40	4.07		0.49	1.0:1.5:2.8		
RSO	0.05	1.44	0.39	0.00	2.72	3.82		0.41	1.0:1.5:2.3		

^1^Main carbohydrate source i.e. M: mung bean starch, R: polished rice + brown rice.

Kind of product i.e. S: snack flake, D: instant drink, SO: instant soup flours.

^2^Calculated by subtraction; ^3^Calculated from formulas.

^4^Conversion factor used: 5.74 for MS; 5.82 for RS; 5.73 for MD; 5.80 for RD; 5.76 for MSO; 5.83 for RSO.

^5^From: WHO/FAO/UNU, 2007; ^6^S:M:P means saturated: monounsaturated: polyunsaturated fatty acids.

Amino acid profile of the flake snack met the FAO/WHO/UNU requirement (World Health Organization 2007). However, concern rested on the branched chain amino acids of isoleucine and valinein the instant cereal drink and soup products (Table 4). The contents of both amino acids were still too low in these products, though they were not expected to be the problem amino acids during formulation. Both drink and soup products contained flakes and powders of different particle size, which might affect non-homogenous sampling. This problem suggests that the instant drink and soup products should be individually packed for one-time preparation, such as one pack for one serving. Bulk packing for several servings could certainly provide inconsistent nutritive values to consumers.

Since the main sources of fat in these formulas were from soybean flour and ground black sesame seed, these products’ fatty acid profiles were high in polyunsaturated fatty acid, P (Table 4). Based on FAO/WHO guidelines, the ratios of P of these products were too high, especially for n-6 (linoleic and linolenic acids), which should contribute only 5–8 kcal/100 kcal (World Health Organization 2003). The fatty acid profile, however, was improved by the addition of rice bran oil, which led to an increase in the monounsaturated fatty acid ratio. Overall, the products still met the FAO/WHO guideline on the percentage of energy contributed from saturated fatty acid that was lower than 8. Limitation of saturated fatty acid consumption reduces the risk of hypercholesterolemia (World Health Organization 2002b).

#### Sensory acceptability

The elderly subjects (219 individuals, 86% female) who voluntarily participated in the sensory central location test had an average age of 71 years with a mode of 65 years. In terms of dental health, 69 percent of the subjects still had more than 20 teeth or dentures, which showed their ability to chew food (Petersen and Yamamoto 2005). Among this group of subjects, 32% still had their own teeth with no dentures. In terms of overall health, 171 subjects acknowledged they had health problems. Hypertension was most common (53%), followed by diabetes mellitus (25%), and hyperlipidemia (15%). In general, each subject had more than one health problem.

Table 5 shows that the subjects liked the general appearance (before tasting) of the products and did not significantly prefer any product over the others, since they all looked similar (as dried flake). However, the scores after tasting were found to be significantly different (*p* < 0.05). All products were acceptable at scores of more than 4.0 (like to like very much). However, both snacks were more accepted than the drinks and soups, and the scores for the drinks and soups were not significantly different (*p* > 0.05). Many subjects mentioned that the flake snack was similar to their traditional crispy snack, which is made from rice. In addition, most of them still had the capacity to chew foods by themselves. Moreover, this group of subjects might not be familiar with drink and soup products, which are common among western style diets. The instant cereal drinks and soups might be more acceptable to urban subjects who are more used to western style diets, as well as in individuals who had chewing problem. Some subjects stated that drinks were too plain and the soups were too salty. Such complaints could be easily solved, since sugar and salt did not contribute to the main nutritive values of the products. Furthermore, reducing salt should be good for their health status since hypertension was a common problem.

**Table 5:** **Sensory overall acceptability scores of the finished products**

Formula ^1^	No. of subject	Overall acceptability ^2,3^	
		Before tasting	After tasting
MS	109	4.08 ± 0.92	4.57^a^ ± 0.66
RS	110	3.95 ± 0.84	4.57^a^ ± 0.58
MD	110	3.80 ± 0.90	4.01^b^ ± 0.94
RD	108	3.88 ± 0.90	4.03^b^ ± 0.88
MSO	109	3.72 ± 0.79	3.91^b^ ± 0.95
RSO	107	3.76 ± 0.88	4.03^b^ ± 0.95

^1^Main carbohydrate sources i.e. M: mung bean starch, R: polished rice + brown rice flours.

Kind of product i.e. S: snack flake, D: instant drink, SO: instant soup.

^2^Rated on 5-point smiley scale: 1, dislike very much; 3, neither like nor dislike; 5, like very much.

^3^Values within the same column without superscript or with the same superscript are not significantly different (p > 0.05).

## Conclusion

By using locally available cereals and legumes, nutritious ready-to-eat food products for elderly persons could be produced in the forms of flake snacks, instant cereal drink and instant soup by using a drum dryer. Rice flour, brown rice flour, and mung bean starch were sources of carbohydrates. Soybean flour and black sesame seed were sources of protein and fat, as well as rice bran oil as a complementary source of fat. The products were balanced in energy distribution from macronutrients and contained good quality protein, as well as being low in saturated fatty acid and free sugar. Most elderly subjects preferred the flake snack products, however all products were sensory acceptable. The developed formulas could be used for production at all industrial scales. The developed products could be a part of Asian’s strategy to deal with its aging society in the near future.

## References

[CR1] AOAC (2006). Official Method of Analysis of AOAC international 2005,1.

[CR2] Arpanantikul M (1999). Formulation of Dietary Fiber-Enriched Bakery containing Guava Pomace and Guava Seeds. MSc Thesis in Food and Nutrition, Faculty of Graduate Studies, Mahidol University.

[CR3] Chernoff R (2006). Geriatric Nutrition: The Health Professional’s Handbook.

[CR4] Elkhalifa AEO, Schiffeler B, Bernhardt R (2005). Effect of fermentation on the functional properties of sorghum flour. Food Chemistry.

[CR5] Food and Agriculture Organization (1997). Carbohydrates in Human Nutrition; Report of a Joint FAO/WHO Expert Consultation, Rome, 14–18.

[CR6] Hagen SR, Frost B, Augustin J (1989). Precolumnphenylisothiocyanatederivatization and liquid chromatography of amino acids in food. Journal of AOAC.

[CR7] Hammond EG, Murphy PA, Johnson LA, Caballero B, Trugo LC, Finglas PM (2003). Soy (soya) beans: properties and analysis. Encyclopedia of Food Sciences and Nutrition.

[CR8] Juliano BO, Perez CM, Komindr S, Banphotkasem S (1989). Properties of Thai cooked rice and noodles differing in glycemic index in noninsulin-dependent diabetics. Plant Foods HumNutr.

[CR9] Lenovich LM, Hui YH (1992). Water activity: microbiology. Encyclopedia of Food Science and Technology.

[CR10] Morley JE, Thomas DR (2007). Geriatric Nutrition.

[CR11] Petersen PE, Yamamoto T (2005). Improving the oral health of older people: the approach of the WHO global oral health programme. Community Dent Oral Epidemiol.

[CR12] Puwasatien P, Raroengwichit M, Sungpuag P, Judprasong K (1999). Thai Food Composition Tables.

[CR13] Sopade PA, Gidley MJ (2009). A rapid in-vitro digestibility assay based on glucometry for investigating kinetics of starch digestion. Starch-Strake.

[CR14] Stone H, Bleibaum RN, Thomas HA (2012). Sensory Evaluation Practices.

[CR15] Tangsermwong T (2005). Glycemic Index of Khanom-tian (KT) in Type 2 Diabetes and Improvement of Plasma Glucose, Serum Lipids and Blood Viscosity after Modification of its Covering Flour. MSc Thesis in Nutrition.

[CR16] Vijaya CSR, Venkatsh S, Arun SM (2007). Grain Drying. Handbook of Industrial Drying.

[CR17] World Health Organization (2002). Active Ageing: A Policy Framework.

[CR18] World Health Organization (2002). Keep Fit for Life, Meeting the Nutritional needs of Older Persons.

[CR19] World Health Organization (2003). Diet, Nutrition and the Prevention of Chronic Diseases; Report of a Joint WHO/FAO Expert Consultation; WHO Technical Report Series no. 916.

[CR20] World Health Organization (2007). Protein and Amino Acid Requirements in Human Nutrition; Report of a Joint WHO/FAO/UNU Consultation; WHO Technical Report Series no.935.

[CR21] World Health Organization (2014). A Healthy Lifestyle. WHO Regional Office for Europe.

